# Patients’ experiences and practices of medication literacy in inflammatory bowel disease: a qualitative study

**DOI:** 10.3389/fphar.2025.1726618

**Published:** 2026-01-06

**Authors:** Wanya Pan, Wenhao Tian, Shuyan Li, Yuan Zhao, Yan He, Yanjie Liu, Xiuqin Feng

**Affiliations:** 1 Department of Nursing, The Second Affiliated Hospital of Zhejiang University School of Medicine (SAHZU), Hangzhou, Zhejiang, China; 2 Zhejiang University School of Medicine, Hangzhou, Zhejiang, China

**Keywords:** health literacy, inflammatory bowel disease, medication literacy, patient education, qualitative study

## Abstract

**Background:**

Medication literacy is critical for the safe and effective use of medications. Patients with inflammatory bowel disease (IBD) face complex and evolving medication regimens. However, little is known about medication literacy in this population. This study aimed to explore the experiences and practices of patients with IBD to characterize their current level of medication literacy.

**Methods:**

A descriptive qualitative research design was adopted. From November 2024 to January 2025, adult patients with IBD were recruited using purposive sampling at a tertiary hospital in China. One-on-one semi-structured interviews were conducted with the patients. All interviews were audio-recorded and transcribed verbatim. The data were analyzed using directed content analysis.

**Results:**

Qualitative analysis of 16 patients revealed the following characteristics across the five core competencies of medication literacy: (1) Accessing medication information: patients were able to obtain information through various channels, primarily healthcare professionals and the internet, but they were not always proactive in seeking it. (2) Understanding medication information: patients knew the names and dosages of their medications, but their understanding of the reasons for long-term use, treatment-plan adjustments, and monitoring indicators was relatively superficial. (3) Communicating medication information: patients were able to talk with healthcare professionals, but their involvement in shared decision-making was limited and they rarely communicated with fellow patients. (4) Evaluating medication information: patients assessed the credibility of information according to the authority of the source, yet found it difficult to judge accuracy on the basis of their own knowledge. (5) Calculating medication information: patients demonstrated strong ability in calculating dosing times and amounts, although they occasionally relied on external tools to ensure accuracy.

**Conclusion:**

Patients with IBD demonstrate a basic level of medication literacy but face challenges across all five dimensions of medication information processing. Findings of this study highlight key components of medication literacy that require development and may provide a foundation for designing targeted assessments and personalized health education programs.

## 1 Introduction

Inflammatory bowel disease (IBD), encompassing Crohn’s disease (CD) and ulcerative colitis (UC), is a chronic, progressive disorder of the gastrointestinal tract with unknown etiology. Although the incidence of IBD has stabilized in Western countries, it is rapidly increasing in newly industrialized countries (Ananthakrishnan et al., 2020), with an estimated 7 million individuals affected by the disease globally (GBD, 2017 Inflammatory Bowel Disease Collaborators, 2020). The condition imposes a substantial burden on global healthcare systems, not only through direct medical expenses but also due to indirect costs, such as decreased productivity, disability and reduced quality of life for affected individuals (Burisch et al., 2023; Burisch et al., 2025).

Patients with IBD often require lifelong pharmacotherapy to control disease activity, sustain remission and prevent complications. Current treatment options span a range of agents, including aminosalicylates, corticosteroids, immunomodulators, biologic agents, and small molecules (Vieujean et al., 2025). The pharmacological regimens prescribed to IBD patients may involve multiple agents administered in different combinations, dosages, frequencies, and routes (Amiesimaka et al., 2024b). Furthermore, therapeutic strategies need to be individualized according to patients’ symptomatic response and treatment tolerability (Lichtenstein et al., 2018). The complexity and variability of these regimens present substantial challenges for patients in using their medications independently.

Studies have shown that medication non-adherence is common among patients with IBD, with rates as high as 50% (Alonso-Abreu et al., 2020). In addition, some patients self-administer corticosteroids and non-IBD medications without consulting gastroenterologist (Mesonero et al., 2021; Lund et al., 2024). These inappropriate medication behaviors increase the risk of adverse outcomes, including disease relapse, loss of drug response, and harmful drug–drug interactions (Ben-Horin and Chowers, 2011; Shah et al., 2020; Fleischmann et al., 2025). Medication literacy has been identified as a key factor in medication adherence and medication self-management, contributing to safer and more effective treatment outcomes (Shi et al., 2019; Wang et al., 2023; Mortelmans et al., 2024).

Medication literacy can be regarded as the application of health literacy in medication use. While closely related to health literacy, it entails distinct skills (Neiva Pantuzza et al., 2022). In 2018, experts with an international perspective defined the medication literacy, that is the degree to which individuals can obtain, comprehend, communicate, calculate and process patient-specific information about their medications to make informed medication and health decisions in order to safely and effectively use their medications (Pouliot et al., 2018). Building upon this foundation, Pantuzza et al. developed a conceptual model of medication literacy, further identifying the specific components for literacy in the context of medication use (Neiva Pantuzza et al., 2022). The core components of this conceptual model include the ability to access, understand, evaluate, communicate, and calculate medication-related information. These five dimensions provide a clear theoretical framework for in-depth investigation of patients’ medication literacy.

The Third Global Patient Safety Challenge “Medication without Harm” strategic plan, launched by the World Health Organization (WHO), proposed that instruments and techniques should be employed to improve patients’ medication literacy and interventions should be developed to promote patients’ knowledge of drug use (Donaldson et al., 2017). Although numerous studies have explored medication literacy among patients with chronic diseases, research specifically focusing on medication literacy in individuals with inflammatory bowel disease remains scarce (Mortelmans et al., 2024; Si et al., 2025; Ming et al., 2025). Previous studies in patients with IBD have primarily focused on topics such as medication knowledge and adherence (Lim et al., 2020; Li et al., 2023; Amiesimaka et al., 2024a). Although a few studies have addressed certain dimensions of medication literacy, their perspectives remain fragmented and fail to comprehensively capture the overall structure and actual level of medication literacy among IBD patients. Therefore, our study aimed to comprehensively understand the medication literacy levels of patients with IBD by exploring their lived experiences and practices through the five core competencies of the medication literacy conceptual model. Addressing this knowledge gap may provide a solid foundation for the development of future assessment tools and intervention strategies.

## 2 Materials and methods

### 2.1 Study design

This study was guided by the conceptual model of medication literacy, developed by multidisciplinary experts through an international Delphi consensus (Neiva Pantuzza et al., 2022). A qualitative descriptive design was adopted to provide a direct and comprehensive description of an experience or event (Doyle et al., 2020). This method is particularly suitable for studying phenomena that are not yet understood, making it ideal for understanding and exploring the experiences and perspectives of patients with IBD regarding medication literacy. We followed the Standards for Reporting Qualitative Research (SRQR) checklist (O'Brien et al., 2014). (Supplementary Table S1).

### 2.2 Study setting and recruitment

The study was conducted from November 2024 to January 2025 at the IBD Center of a tertiary-level hospital in Hangzhou, Zhejiang Province, China. The center provides services for patients with IBD, including medical treatment, health education, and follow-up care. Participants who met the inclusion criteria were purposively sampled from inpatient wards and outpatient clinics. In addition, a maximum variation sampling strategy was adopted to ensure sample heterogeneity based on patients’ age, gender, educational levels, disease type, and duration of disease. The sample size was determined based on the principle of data saturation. Data collection was concluded when no new information emerged from subsequent interviews or analyses, and when existing themes remained stable without further modification (Hennink and Kaiser, 2022). Two additional patients were interviewed to verify data saturation.

### 2.3 Inclusion and exclusion criteria

Inclusion criteria were as follows: (1) patients met the diagnostic criteria of the 2023 Chinese Guidelines for the Diagnosis and Treatment of UC and CD (Inflammatory Bowel Disease Group of Chinese Society of Gastroenterology of Chinese Medical Association and Inflammatory Bowel Disease Quality Control Center of China, 2024a; Inflammatory Bowel Disease Group of Chinese Society of Gastroenterology of Chinese Medical Association and Inflammatory Bowel Disease Quality Control Center of China, 2024b); (2) age ≥18 years and disease duration ≥6 months; (3) had used at least one type of medication for the treatment of IBD; (4) native language is Chinese, with basic communication, expression, and comprehension abilities; and (5) participating voluntarily. Exclusion criteria were as follows: (1) patients with other chronic conditions requiring long-term medication (e.g., hypertension or diabetes); (2) patients with severe life-threatening diseases (e.g., upper gastrointestinal bleeding, malignancies); and (3) patients with severe cognitive impairment or mental illness.

### 2.4 Data collection

This study was guided by the five subdimensions of the medication literacy conceptual model as its theoretical framework. Based on a literature review, a semi-structured interview outline was developed through consultations with experts in clinical gastroenterology, nursing, and pharmacy related to IBD. Prior to the formal interviews, two eligible participants were selected through convenience sampling to conduct a pilot interview. The outline was revised and refined based on feedback from these pilot interviews to create the final version. The formal interview outline can be seen in Supplementary Table S2.

Each interview was conducted by the same researcher (WP) to ensure consistency. Data was collected using a semi-structured one-on-one interview method offline. Interviews were arranged at times convenient for patients to ensure continuity and minimize interruptions. They were conducted in quiet, comfortable settings to reduce anxiety and encourage open communication. Before the formal interviews, researcher established rapport with participants, explained the purpose, methods, procedures, and estimated duration of the interview, and obtained informed consent. During the interviews, researcher followed the interview guide and audio-recorded the entire conversation using a digital voice recorder. The order and phrasing of questions were adjusted in real time based on their responses. Probing, paraphrasing, and clarification techniques were used when appropriate to encourage participants to express their views and experiences fully. While ensuring the smooth progress of the interview, researcher closely observed and promptly recorded their nonverbal behaviors, including facial expressions, tone of voice, and body movements.

### 2.5 Data analysis

Within 24 h after each interview, the audio recordings were transcribed verbatim by the researcher (WP). The transcribed transcripts were reviewed by another researcher (WT) through cross-checking with the original audio recordings. Then the transcripts were returned to the participants for verification to ensure the accuracy and authenticity of the data. Two researchers (WP and WT) conducted a directed content analysis of the interview data using NVivo 20 software. This analytic approach is well-suited for further developing or extending existing theories or research on a particular phenomenon (Hsieh and Shannon, 2005). The specific analysis process is as follows: (1) the five subdimensions of the medication literacy conceptual model were used as the initial coding categories, and each category was assigned an operational definition; (2) they repeatedly read the interview transcripts line by line to extract meaningful units relevant to the research content; (3) they categorized and coded the meaningful units using the initial coding categories; (4) content that could not be classified using the initial coding categories was assigned new codes, and the existing coding scheme was modified when necessary; (5) after completing all coding, the codes were grouped into new categories and subcategories based on their conceptual relationships; and (6) the categories then were organized and conceptualized into themes. Discrepancies were resolved through discussion within the research team until consensus was reached.

In this study, all participants were transcribed verbatim in Chinese, and the data analysis was based on the original Chinese transcripts. During the preparation of the English manuscript, a bilingual team member (WP) with expertise in IBD and qualitative research translated representative participant quotes into English. These translations were then cross-checked by another bilingual author (SL) with similar expertise to ensure that the meaning and context of the original Chinese expressions were preserved to the greatest extent possible.

### 2.6 Rigor and reflexivity

The rigor of this study was ensured through four criteria: credibility, transferability, dependability, and confirmability (Lincoln and Guba, 1985). To ensure credibility, participant feedback on the research findings was solicited. Transferability was supported by providing a detailed description of the participants’ backgrounds, the research context, and the interaction process, allowing the findings to be applicable in other settings. Dependability was maintained through the establishment of a complete audit trail, with thorough documentation of the data analysis process. To enhance confirmability, the findings were grounded in the original data, minimizing the influence of researcher bias. Researchers received systematic training in qualitative research methodology. In addition, the first author kept a reflexive journal to record personal thoughts, assumptions, and potential biases, which were continuously discussed with the research team to strengthen the overall trustworthiness of the study.

### 2.7 Ethical considerations

This study was approved by the Ethical Committee of the Second Affiliated Hospital, Zhejiang University School of Medicine on 11 November 2024 (approval number 2024-1392). Written informed consent was obtained from all participants. This study placed strong emphasis on confidentiality and privacy. Participants were anonymized by assigning numerical codes based on the interview sequence, ensuring that no data could be linked to individual identities. All collected data were used solely for this study to protect the privacy of participants.

## 3 Results

### 3.1 Characteristics of participants

A total of 16 patients with IBD were interviewed, with disease duration ranging from 6 months to 26 years. The sample included 8 patients with CD and 8 with UC, comprising 8 males and 8 females. Participants ranged in age from 18 to 60 years. Each interview lasted between 30 and 50 min. See Table 1 for details. Table 2 summarizes the relationships between the five dimensions of medication literacy and their corresponding subthemes.

**TABLE 1:** Characteristics of participants (n = 16).

Code	Gender	Age (years)	Education	Disease type	Duration of disease (years)	Place of residence	Current medication regimen	Medications previously administered
N1	Female	24	Bachelor’s degree	CD	1	Urban areas	Infliximab	Mesalazine MP
N2	Male	26	Master’s degree	UC	10	Urban areas	Vedolizumab	Mesalazine MP
N3	Male	54	Primary school	UC	3	Rural areas	Vedolizumab Mesalazine	None
N4	Female	52	Bachelor’s degree	CD	8	Urban areas	Infliximab	Mesalazine, azathioprine
N5	Female	36	Junior high school	CD	15	Rural areas	Infliximab	Mesalazine, infliximab
N6	Male	37	Bachelor’s degree	UC	11	Urban areas	Vedolizumab	Mesalazine
N7	Male	33	Associate degree	CD	5	Rural areas	Infliximab	Mesalazine
N8	Female	40	Bachelor’s degree	CD	26	Urban areas	Infliximab Azathioprine	Mesalazine, prednisone, sulfasalazine
N9	Female	35	Master’s degree	UC	7months	Urban areas	MP Infliximab Vedolizumab	Mesalazine
N10	Male	34	Master’s degree	CD	2	Urban areas	Infliximab	None
N11	Female	28	Master’s degree	UC	3	Urban areas	Mesalazine	Mesalazine
N12	Male	22	Bachelor’s degree	CD	6months	Rural areas	Infliximab	MP, azathioprine
N13	Female	43	Primary school	UC	1	Rural areas	Infliximab	Mesalazine, prednisone Vedolizumab
N14	Female	38	Junior high school	CD	4	Rural areas	Ustekinumab	Mesalazine, MP
N15	Male	60	Senior high school	UC	18	Urban areas	Vedolizumab	Mesalazine, MP
N16	Male	18	Senior high school	UC	1	Rural areas	Ustekinumab	Mesalazine, infliximab

Abbreviations: CD, Crohn’s disease; UC, ulcerative colitis; MP, methylprednisolone.

**TABLE 2:** Themes and subthemes of medication literacy in IBD patients.

Themes	Subthemes
1. Ability to access medication information	1.1 channels of acquisition
	1.2 proactiveness in information acquisition
	1.3 content of acquired information
2. Ability to understand medication information	2.1 The importance of adhering to proper medication use
	2.2 content of key medication information
	2.3 individualized medication use
	2.4 adjustment of medication regimens
	2.5 content of medication-related monitoring indicators
3. Ability to communicate medication information	3.1 communication with healthcare professionals
	3.2 participation in shared decision-making on medication
	3.3 communication with fellow patients
4. Ability to evaluate medication information	4.1 reliability of medication information
	4.2 physical symptom changes
	4.3 abnormal test results
5. Ability to calculate medication information	5.1 storing and calculating medication-related numerical information

### 3.2 Theme 1: Ability to access medication information

#### 3.2.1 Channels of acquisition

Patients accessed information about IBD medications through multiple channels, including printed materials, online platforms, health education from medical professionals, and peer communication with other patients. However, most patients primarily relied on medical professionals and the internet as their main sources. Only a few patients used digital tools with limited functions, such as those that offer only consultation and inquiry services, to obtain information.

“In our (doctor-patient) group chat, doctors would post some tweets about new treatment methods, and the other patients would share them, have a look, that kind of thing.” (N2).

“I searched online and found some articles written by others, or like, some videos posted by doctors or something. I’ve watched quite a lot of videos. Then I also bought a book.” (N10).

#### 3.2.2 Proactiveness in information acquisition

Some older or less-educated patients reported that they had never actively sought medication-related information and had remained passive recipients. They placed complete trust in, and relied entirely on, the health education provided by physicians and other healthcare professionals.

“I did not really look into the medicine info, ‘cause I felt like. after the symptoms got better, just following what the doctor said would be fine.” (N3).

“I did not pay attention to it (medication information), you know. Whatever the doctor said, that’s what I did.” (N13).

Most patients reported that although they possessed the ability to actively obtain medication-related information, they did not do so consistently or intentionally. Instead, they tended to search for information only when they had a clear and immediate need—particularly during the early stages of diagnosis or periods of disease exacerbation. As their condition stabilized and daily life returned to normal, patients were more likely to engage with relevant information passively, even when it was of interest, unless they encountered specific questions or practical concerns.

“When I was in the hospital, that was the first time I found out I had this disease, so I focused on learning about all that stuff—like the medication and everything. But now that I’m back to normal work and life, I only look at something if it really catches my attention. I do not, like, actively search for that kind of info anymore like I did back then.” (N9).

#### 3.2.3 Content of acquired information

When seeking medication-related information, patients focused on various aspects, including side effects, efficacy, usage precautions, and relevant laboratory indicators. Among these, side effects and efficacy were of particular concern. Especially during the stage of treatment decision-making, when multiple therapeutic options were available, patients often weighed the benefits and risks by reviewing such information to make the most appropriate choice for their individual condition.

“I first picked some treatments that I thought fit me. Then I’d also check the effects of the medicine myself, but like. mostly just to see if there’s anything really negative, like strong side effects or stuff like that. After looking into it, I thought, okay, I can probably take this one (Infliximab).” (N10).

Currently, medications used to treat IBD rarely result in a complete cure and are often associated with adverse effects. In this context, some patients in long-term remission expressed concern about the sustained effectiveness of current therapies. As a result, they actively focused on information about drugs they have not yet used, including alternative or emerging treatments, in the hope that future breakthroughs may offer a potential cure.

“Also, I do pay attention to some new medicines, ‘cause I do not know. like, after using this one for a long time, will it develop resistance or something. It’d be best if there’s a new drug that could totally cure it (CD). I trust medicine—I believe there’ll be hope in the future.” (N4).

### 3.3 Theme 2: Ability to understand medication information

#### 3.3.1 The importance of adhering to proper medication use

A few participants recognized early after diagnosis that IBD is currently incurable and requires long-term pharmacological management. They appeared to have a clear understanding of the importance of consistent and regular medication adherence.

“I know this disease cannot be cured or anything now, and you have to take medicine long-term.” (N9).

However, some participants lacked a full understanding of the importance of long-term and consistent medication use, mistakenly believing that the disease could be cured in a short time. Some had even discontinued their medications without medical guidance. It was not until symptoms recurred that they gradually came to recognize the critical role of sustained and appropriate medication use in maintaining remission.

“I took mesalazine for about a month, and it worked really well—no more mucus or blood in the stool. Back then I did not know, I thought it was cured, so I just stopped it on my own. Then like a week later, there was a bit of blood again. Maybe the doctor told me not to stop, but I probably did not understand clearly, ‘cause it was my first time using it, and I did not know this kind of thing.” (N3).

#### 3.3.2 Content of key medication information

Although a few participants lacked a clear understanding of their medication regimens even after years of living with the disease, the majority demonstrated a high level of awareness regarding their prescribed medications. Most were able to clearly and accurately describe key information such as the drug names, precautions, dosages, and administration methods.

“At the beginning I was taking 6 tablets of methylprednisolone, reducing one tablet per week. I took it for about a month. Then I started taking azathioprine together with methylprednisolone. Azathioprine was like one tablet a day, something like that.” (N12).

In addition to basic medication information, most participants had a preliminary understanding of adverse effects, mechanisms of action, and therapeutic purposes. However, their knowledge in these areas was generally superficial, often limited to vague concepts, and lacked the depth needed for systematic and accurate articulation.

“Taking medicine comes with all kinds of side effects. It lowers your immunity, makes it easier to get viral infections, and also things like eczema or other skin problems.” (N10).

“I looked into steroids too. It can shut down the whole immune system, that kind of mechanism.” (N11).

#### 3.3.3 Individualized medication use

Most participants recognized that significant individual differences exist in medication therapy. They believed that both efficacy and adverse effects vary from person to person, and that treatment decisions should not be based blindly on others’ experiences. Instead, patients emphasized the importance of choosing medications that are suitable for their own conditions and responses.

“Taking medicine is also a kind of overall consideration. If others used it and did well, then maybe it's good—but that does not mean it’ll 100% work for you. Whether it helps your gut or not, you’ll only know after you try it yourself.” (N7).

Some participants illustrated individual variability in drug response by sharing their own experiences. They expressed an understanding of, and acceptance toward, medication adjustments made based on their personal reactions.

“Everyone reacts differently to medicine. Like for me, I got allergic to Remicade, so I could only push it really slowly—like 15 mL per hour.” (N9).

#### 3.3.4 Adjustment of medication regimens

Adjusting medication regimens is a common practice in the treatment of IBD. In this study, most participants reported having experienced medication adjustments during their treatment journey. They viewed such adjustments as a routine therapeutic strategy in response to inadequate efficacy or changes in disease condition. Some participants also offered personal perspectives on the necessity and appropriateness of switching medications, drawing from their own or others’ treatment experiences.

“I feel like switching meds is a way of adjusting the treatment. Like, if you need to quickly control really serious symptoms, then for sure you need stronger drugs. Like that time, I was on methylprednisolone plus azathioprine—that was because it was pretty serious. Later, when the inflammation went down, then I could switch to something milder, so it’s less harmful to the body.” (N12).

#### 3.3.5 Content of medication-related monitoring indicators

Most participants recognized that regular monitoring of relevant indicators is important for assessing disease activity, evaluating medication efficacy, and detecting adverse effects. They were generally familiar with the required tests during pharmacological treatment and were able to accurately describe the purpose, content, and frequency of these examinations. This reflected a clear understanding of the monitoring process and the scheduling of related procedures.

“These test indicators are important—they can show whether the medication is starting to not work anymore.” (N10).

“Azathioprine has some side effects, like low white blood cell count and stuff. It might also affect your liver and kidneys, so you need to do blood tests regularly—like blood routine, ESR, liver and kidney function tests. If those are all fine, then there’s nothing to worry about.” (N12).

Despite repeated emphasis from physicians, some participants underestimated the importance of regular monitoring due to symptom relief and personal inertia. Their level of attention to follow-up examinations had declined, and they did not strictly adhere to medical advice, exhibiting a degree of inconsistency in actual practice.

“I just feel it’s a hassle, and sometimes I’m kinda lazy. If I feel better, I do not wanna check those indicators. Like calprotectin—I have not checked it in a long time. The doctor suggested it, but I did not do it.” (N6).

Some participants demonstrated a good understanding of routine monitoring indicators and were able to interpret changes in these indicators reasonably in relation to their own disease condition.

“A few times in a year, my calprotectin suddenly goes up—it shows the inflammation is still kind of acting up.” (N7).

However, some participants showed limited understanding of monitoring indicators. Even after reading the explanatory notes in the test reports, they still struggled to accurately grasp the specific meanings of various indicators.

“One is the drug concentration check, and the other is those blood test indicators. These two seem to be kind of related. I read the instructions myself, but I did not really understand it very well.” (N10).

### 3.4 Theme 3: Ability to communicate medication information

#### 3.4.1 Communication with healthcare professionals

Many participants reported that when encountering abnormal situations or issues they could not resolve on their own during medication use, they were generally proactive in consulting their primary physicians or other healthcare professionals to obtain professional information and guidance.

“If there’s something I do not understand, I can just ask. I used to have questions, but after asking the doctor, now I basically get it.” (N4).

“Last time I had pneumonia and just recovered, but the time to take the injection was coming. So I asked the guy (doctor’s assistant) who helped me book it—like, can I still get the shot in this situation?” (N13).

#### 3.4.2 Participation in shared decision-making on medication

A few participants were not merely passive recipients of physician-prescribed treatment plans but actively engaged in discussions regarding medication selection and adjustments. This suggests that they possessed, to some extent, the ability to integrate personal experiences with medical advice in the decision-making process.

“Later I started using biologics, for almost 2 years, but my skin problems got worse and worse. At that time I could not find a good dermatologist, so I talked to my main doctor about stopping the medicine.” (N8).

#### 3.4.3 Communication with fellow patients

Some participants reported that, in addition to interacting with healthcare professionals, they also engaged in communication with fellow patients through both online and offline channels. These discussions primarily focused on sharing medication experiences, which helped deepen their understanding of the medications.

“I joined a WeChat group for university students. People usually chat in there about their health condition, meds, stuff like that.” (N12).

Although some participants were capable of communicating with fellow patients, they perceived the value of such discussions as limited and expressed a preference for consulting physicians directly when faced with medication-related issues.

“I did join a (WeChat) group, but do not really talk in it. If I have questions, I just ask the doctor directly. Talking among ourselves does not really help that much.” (N3).

### 3.5 Theme 4: Ability to evaluate medication information

#### 3.5.1 Reliability of medication information

Most participants relied primarily on the perceived authority of the information source when assessing the authenticity and professionalism of medication-related content. They generally considered information from official institutions or professional medical platforms to be more reliable and were unlikely to question content provided through these channels.

“I think the first thing is to check how reliable the public account is. Those random accounts can easily make stuff up or repost unverified info. But generally the more official ones, the websites, should be more accurate.” (N9).

“I leave professional stuff to the professionals. I will not question what the doctor tells me.” (N16).

Although most participants tended to trust information provided by doctors or professional institutions, some did not rely solely on a single source. When receiving medication information from their doctors, they actively sought verification through other channels to confirm the accuracy and consistency of the content.

“I’ll double-check. I do not just accept what the doctor says right away. I might go and look things up myself, and if what I find is more or less the same as what he said, then I can trust it.” (N1).

#### 3.5.2 Physical symptom changes

During the course of medication use, some participants were able to make preliminary judgments about reduced drug efficacy or potential adverse reactions by observing and perceiving changes in their physical condition.

“When I took oral and suppository mesalazine, I got all itchy and my face swelled up. But as soon as I stopped the medicine, all the symptoms went away in 2 days. So yeah, clearly allergic to it.” (N9).

“I clearly felt my body was bad during the time I was on metronidazole. The frequency and shape of my stool were just not right.” (N11).

However, some participants had difficulty understanding the causes of their symptoms and determining whether their physical discomfort was medication-related.

“After using this biologic, I felt kind of tired. Is that a side effect?” (N15).

#### 3.5.3 Abnormal test results

Many patients were concerned about the results of tests conducted during medication use. They were able to use their existing knowledge or external cues to determine whether the test results are within the normal range.

“The calprotectin is within the normal range, but it’s not the best result.” (N11).

In addition to evaluating whether individual indicators are abnormal, some patients also expressed concern about the relationships among multiple test results. They attempted to infer their disease status or treatment response based on fluctuations in these indicators.

“I noticed when the Remicade level of Infliximab was around 10, my calprotectin and blood indicators were all pretty good. I’m especially sensitive to calprotectin—it stays below 200 when things are okay.” (N8).

### 3.6 Theme 5: Ability to calculate medication information

#### 3.6.1 Storing and calculating medication-related numerical information

Participants generally demonstrated a high level of attention to core treatment parameters such as medication timing and dosage. They were able to clearly identify and prioritize the memorization of this key information. When faced with the risk of forgetting, many employed personalized external aids—such as notes, reminders, or digital tools—to record medication details and ensure accuracy. Based on this information, they were able to perform calculations to determine precise dosing schedules and quantities.

“I remembered how often I need to go get the injection. I just counted it out week by week on the calendar, so I know when it’s time to go.” (N13).

“At first I was taking 4 steroid pills, and reduced by half a pill each week. So after a week it went down to 3 and a half.” (N14).

## 4 Discussion

Guided by the conceptual model of medication literacy, this study aimed to explore the experiences and practices of patients with IBD related to medication literacy and to assess their overall level of medication literacy. The results revealed that patients exhibited deficiencies across all five dimensions of medication literacy, highlighting the need for targeted interventions to help them overcome barriers in accessing, understanding, communicating, evaluating, and calculating medication-related information.

### 4.1 Ability to access medication information

Findings from this study indicated that patients with IBD primarily accessed medication information through healthcare professionals and online sources, which aligns with the conclusions of previous scoping reviews (Norouzkhani et al., 2023; Ni et al., 2024). This is because patients generally prefer communication with IBD physicians or nurses, reflecting their recognition of healthcare professionals’ authority and credibility (Ni et al., 2024). At the same time, online platforms such as WeChat official accounts have become increasingly important sources of information, valued for their convenience and richness of content (Karadag et al., 2022). Although digitally tailored tools for IBD have demonstrated growing potential in supporting patients in recent years (Asadi et al., 2024; Lin et al., 2025), patients in this study used such functionalities primarily for online consultations and inquiries. Overall utilization was low, likely reflecting established care-seeking habits, and most patients continued to prefer face-to-face communication with healthcare professionals. None of the participants reported using self-management applications with interactive data-management capabilities. This gap underscores the need to develop high-quality, culturally adapted digital tools that integrate authoritative information into routine care to support self-management.

In addition, this study revealed that patients’ information-seeking behaviors and needs changed with the progression of the disease. Most patients had the ability to actively search for information, especially during the initial diagnosis or disease flare-ups; as symptoms improved and life stabilized, they tended to shift toward more passive information-seeking methods (Lai et al., 2019). Frequent active searches are often associated with higher perceived stress and symptoms of post-traumatic stress, while better health-related quality of life tends to reduce the need for active information seeking (Pittet et al., 2016). Older patients and those with lower educational levels relied more on physicians and were less likely to actively seek information, suggesting that age and educational background may be major limitations to autonomous information acquisition. Our findings revealed that patients preferred to obtain information about medication efficacy and potential side effects, which may reflect their heightened focus on the expected benefits and risks of medications, particularly during the treatment decision-making phase (Thompson et al., 2016; Daher et al., 2019). When patients realize that current medications cannot provide a cure, particularly those in remission, they often begin to focus on advancements in new drug research and place hope in innovative therapies for the possibility of a cure. This result is consistent with previous studies (Lesnovska et al., 2014; Pittet et al., 2016). In light of this, healthcare professionals may need to enhance the dissemination of high-quality online information and digital health education, while also providing targeted, medication-related information and education tailored to individual patients’ conditions and needs.

### 4.2 Ability to understand medication information

Adherence to prescribed medication is critical for maintaining disease remission. This study revealed that some patients misunderstood the long-term nature of treatment and believed that medication could be discontinued once symptoms resolved. This observation is consistent with previous studies conducted in Switzerland and Japan (Pittet et al., 2016; Watanabe et al., 2022). Resolution of symptoms often leads patients to mistakenly believe that the disease is cured, thereby reducing adherence to long-term treatment. Healthcare providers should therefore emphasize the necessity of continuous, regular medication use and correct patients’ misconceptions through effective risk communication. Although participants were familiar with basic information such as medication names, usage, and dosages, their understanding of indications and potential adverse effects remained limited, primarily due to a lack of relevant knowledge (Shao et al., 2023; Xie et al., 2023). The majority of participants recognized that medication efficacy and side effects vary among individuals, which is consistent with the results of a cross-sectional study (Shao et al., 2023). A correct understanding of personalized treatment helps patients adopt a rational perspective on medication efficacy and avoid blindly following others’ medication experiences. Most patients were also receptive to adjustments in their treatment plans, viewing medication changes as a common strategy. Clinicians should provide comprehensive medication education and enhance follow-up to help patients better understand the treatment plan, thereby improving therapeutic outcomes.

Patients generally acknowledged the importance of monitoring medication-related indicators, considering monitoring results crucial for treatment adjustments. However, despite reminders from physicians to undergo follow-up examinations, patient compliance still depends on individual initiative, especially during remission periods when some patients tend to neglect regular monitoring. Enhancing patients’ understanding of the role of monitoring in disease surveillance and relapse prevention may increase their awareness of the importance of regular follow-up, thereby reducing potential risks (van der Mee et al., 2024).

### 4.3 Ability to communicate medication information

The results of this study indicated that most patients were able to proactively communicate with healthcare professionals regarding medication-related issues, and some patients were even involved in shared decision-making about their medication. This reflected their level of awareness about their treatment plan and their ability to clearly express personal preferences and values. Such communication skills lay the foundation for the shared decision-making model advocated by clinical guidelines (Rubin et al., 2025). However, previous studies have pointed out that, despite patients’ willingness and ability to communicate, differences in treatment perspectives between patients and clinicians hinder the fulfilment of patients’ communication needs, thereby contributing to communication gaps (Rubin et al., 2021; Watanabe et al., 2022). Furthermore, patients often sought information and emotional support through peer interactions (O'Leary et al., 2020), a behavior that also observed in patients with various chronic conditions (Reidy et al., 2024). Given the long-term uncertainty and substantial psychological burden associated with chronic diseases such as IBD, patients often seek experiential knowledge and emotional resonance from others with similar lived experiences (Thompson et al., 2022). In summary, healthcare professionals should strengthen personalized health education and patient-provider interactions, while also encouraging patients to participate in support groups to promote interpersonal communication and the establishment of a social support network.

### 4.4 Ability to evaluate medication information

In this study, patients demonstrated limited capacity for critical assessment regarding the quality of information. Most patients did not evaluate the content of information itself but instead overly relied on the credibility of the information source, often accepting information from healthcare professionals or authoritative organizations without question. A study on eHealth literacy also supported this finding (Chang et al., 2021). This highlights the need to improve the quality of online information and even introduce professional content certification mechanisms as essential measures to ensure that patients receive accurate and reliable IBD medication information (Yoon et al., 2019). By accessing medication knowledge through high-quality official websites or medical platforms, patients are able to gradually identify inaccurate or misleading information, thereby enhancing their ability to filter and evaluate information. Notably, a small number of participants demonstrated strong critical thinking skills, cross-checking information from multiple sources, thus providing a model for other patients to improve their medication literacy. Additionally, although most patients were able to determine whether test results indicated abnormalities, they remained confused about whether new symptoms were related to their medication. Therefore, healthcare professionals should strengthen education on adverse drug reactions and their recognition methods to help patients improve their ability to identify and respond to medication-related issues early, ensuring safer and more effective treatment (Thomas et al., 2020; Wang et al., 2024).

### 4.5 Ability to calculate medication information

Numeracy plays a crucial role in preventing medication errors and supporting effective self-management by helping patients accurately process medication-related numerical information (Golbeck et al., 2005; Pouliot et al., 2018). In this study, the majority of participants were able to recall and calculate key medication information, such as dosage timings and schedules, ensuring accurate medication use. The ability to correctly process numerical information relies on the foundation of numerical recognition and understanding of quantitative data. Patients must first grasp the meaning of this information before they can develop higher-level computational skills (Golbeck et al., 2005). A health literacy study on nonsteroidal anti-inflammatory drug (NSAID) labeling pointed out that clinicians often spend limited time explaining numerical information (such as dosage or lab values) and its clinical significance, which becomes a major barrier to patients’ understanding of numerical information (Jang et al., 2019). Therefore, healthcare professionals should regularly implement targeted health education interventions to improve patients’ medication-related numerical literacy, guiding them to accurately calculate and apply these values, thereby enhancing treatment outcomes.

### 4.6 Implications for policy and practice

This study revealed the lived experiences and real-world practices of medication literacy among patients with IBD, highlighting existing deficiencies in this population and offering new perspective on patients’ medication self-management. The findings underscored the necessity of developing and validating scientifically robust assessment instruments of medication literacy and targeted health-education programs. It is recommended that standardized medication literacy screening be incorporated into routine clinical pathways to systematically identify patients’ supportive needs. Moreover, in light of gaps within current clinical support systems, healthcare professionals should strengthen patient-centered communication and educational strategies, provide clear and concise medication guidance, and integrate medication literacy enhancement measures into daily care to improve medication safety and medication adherence.

### 4.7 Limitations

Several limitations should be acknowledged. Firstly, this study was designed as a single-center investigation, with participants recruited from the IBD center of a tertiary hospital in a major city in China. Although a maximum variation sampling strategy was employed to capture diverse perspectives, the study’s setting and sample source may limit the inclusion of patients with a wider range of cultural backgrounds, socioeconomic statuses, and healthcare experiences. This, in turn, could impact the generalizability and broader applicability of the findings. Secondly, while some participants described evolution of their medication experiences and perceptions during the interviews, the cross-sectional design of this study made it difficult to explore the trajectories and underlying causes of these changes. As a result, the dynamic nature of medication literacy among IBD patients could not be fully elucidated. Future research should consider conducting multi-regional, multi-center, and cross-cultural longitudinal qualitative studies, including patients from diverse regions and healthcare levels, to expand both the depth and breadth of the research, thereby enhancing the transferability and applicability of the findings.

## 5 Conclusion

This study adopted the conceptual model of medication literacy as its theoretical framework to systematically explore the real-world experiences, competency performance, and existing challenges of patients with IBD across five dimensions: accessing, understanding, communicating, evaluating, and calculating medication-related information. Patients demonstrated a certain level of medication literacy. However, they showed deficiencies across all five dimensions of medication literacy, particularly in critically evaluating the authenticity and reliability of medication information. Moreover, their medication literacy may be influenced by multiple factors, including individual characteristics, social environment, disease characteristics, and healthcare systems. Further research is needed to gain a more comprehensive understanding of their experiences, perspectives, and influencing factors in terms of medication literacy by including patients from different regions and cultural backgrounds. In addition, it is essential to develop assessment tools that can effectively measure the level of medication literacy among patients with IBD. By combining actual levels and potential influencing factors, personalized intervention strategies can be developed to improve medication literacy skills in a targeted manner.

## Data Availability

The original contributions presented in the study are included in the article/Supplementary Material, further inquiries can be directed to the corresponding author.
