# Supplementary material for: Patients’ experiences and practices of medication literacy in inflammatory bowel disease: a qualitative study
Source: Front Pharmacol. 2026 Jan 6;16:1726618. doi: 10.3389/fphar.2025.1726618 (PMC12815703; doi:10.3389/fphar.2025.1726618)
Supplement: Supplementary file 1 [file Supplementaryfile1.docx]

Supplementary Material

**Supplementary S1:**

**Standards for Reporting Qualitative Research (SRQR).**

| **No.** | **Topic** | **Item** | **Article sections** |
| --- | --- | --- | --- |
|  | **Title and abstract** |  |  |
| S1 | Title | Concise description of the nature and topic of the study; identifying the study as qualitative or indicating the approach (e.g., ethnography, grounded theory) or data collection methods (e.g., interview, focus group) is recommended | Title |
| S2 | Abstract | Summary of key elements of the study using the abstract format of the intended publication; typically includes background, purpose, methods, results, and conclusions | Abstract |
|  | **Introduction** |  |  |
| S3 | Problem formulation | Description and significance of the problem/phenomenon studied; review of relevant theory and empirical work; problem statement | Introduction |
| S4 | Purpose or research question | Purpose of the study and specific objectives or questions | Introduction |
|  | **Methods** |  |  |
| S5 | Qualitative approach and research paradigm | Qualitative approach (e.g., ethnography, grounded theory, case study, phenomenology, narrative research) and guiding theory if appropriate; identifying the research paradigm (e.g., postpositivist, constructivist/interpretivist) is also recommended; rationale | Study design |
| S6 | Researcher characteristics and reflexivity | Researchers’ characteristics that may influence the research, including personal attributes, qualifications/experience, relationship with participants, assumptions, and/or presuppositions; potential or actual interaction between researchers’ characteristics and the research questions, approach, methods, results, and/or transferability | Data collection, Data Analysis, Rigor and reflexivity |
| S7 | Context | Setting/site and salient contextual factors; rationale | Study Setting and Recruitmen |
| S8 | Sampling strategy | How and why research participants, documents, or events were selected; criteria for deciding when no further sampling was necessary (e.g., sampling saturation); rationale | Study Setting and Recruitment, Inclusion and Exclusion Criteria |
| S9 | Ethical issues pertaining to human subjects | Documentation of approval by an appropriate ethics review board and participant consent, or explanation for lack thereof; other confidentiality and data security issues | Ethical Considerations |
| S10 | Data collection methods | Types of data collected; details of data collection procedures including (as appropriate) start and stop dates of data collection and analysis, iterative process, triangulation of sources/methods, and modification of procedures in response to evolving study findings; rationale | Data collection |
| S11 | Data collection instruments and technologies | Description of instruments (e.g., interview guides, questionnaires) and devices (e.g., audio recorders) used for data collection; if/how the instrument(s) changed over the course of the study | Data collection |
| S12 | Units of study | Number and relevant characteristics of participants, documents, or events included in the study; level of participation (could be reported in results) | Characteristics of participants, Table1 |
| S13 | Data processing | Methods for processing data prior to and during analysis, including transcription, data entry, data management and security, verification of data integrity, data coding, and anonymization/deidentification of excerpts | Data Analysis |
| S14 | Data analysis | Process by which inferences, themes, etc., were identified and developed, including the researchers involved in data analysis; usually references a specific paradigm or approach; rationale | Data Analysis |
| S15 | Techniques to enhance trustworthiness | Techniques to enhance trustworthiness and credibility of data analysis (e.g., member checking, audit trail, triangulation); rationale | Rigor and reflexivity |
|  | **Results/findings** |  |  |
| S16 | Synthesis and interpretation | Main findings (e.g., interpretations, inferences, and themes); might include development of a theory or model, or integration with prior research or theory | Results |
| S17 | Links to empirical data | Evidence (e.g., quotes, field notes, text excerpts, photographs) to substantiate analytic findings | Results |
|  | **Discussion** |  |  |
| S18 | Integration with prior work, implications, transferability, and contribution(s) to the field | Short summary of main findings; explanation of how findings and conclusions connect to, support, elaborate on, or challenge conclusions of earlier scholarship; discussion of scope of application/generalizability; identification of unique contribution(s) to scholarship in a discipline or field | Discussion |
| S19 | Limitations | Trustworthiness and limitations of findings | Limitations |
|  | **Other** |  |  |
| S20 | Conflicts of interest | Potential sources of influence or perceived influence on study conduct and conclusions; how these were managed | Conflict of Interest |
| S21 | Funding | Sources of funding and other support; role of funders in data collection, interpretation, and reporting | Funding |

**Reference:** O'Brien BC, Harris IB, Beckman TJ, Reed DA, Cook DA. Standards for reporting qualitative research: a synthesis of recommendations. Academic Medicine, Vol. 89, No. 9 / Sept 2014 DOI: 10.1097/ACM.0000000000000388

**Supplementary S2：The formal interview outline**

| **1. Introductory question** | (1) Could you describe in detail your experiences with medication therapy after becoming ill? |
| --- | --- |
| **2. Questions on the dimensions of medication literacy** | **2.1 Accessing**  (2) What aspects do you pay attention to during medication use? How do you obtain this medication information? |
|  | **2.2 Understanding**  (3) Do you understand what the medication information you obtain means? How do you interpret or make sense of it? |
|  | **2.3 Evaluating**  (4) How do you determine whether the medication information is accurate and reliable? How do you analyze whether this information is applicable to you? |
|  | **2.4 Communicating**  (5) With whom have you discussed medication-related matters? What aspects were discussed? |
|  | **2.5 Calculating**  (6) What aspects of medication use involve numbers or calculations? How did you handle them? |
| **3. Exploratory questions** | (7) How did you use medication information to help you use medications correctly and safely? |
|  | (8) What difficulties did you encounter during medication use? How did you resolve them? |
